# Georgia State Dental Society

**Published:** 1886-08

**Authors:** 


### PORCEEDINGS OF SOCIETIES.

### Georgia State Dental Society.

EIGHTEENTH ANNUAL MEETING, MAY, 1886.

(Reported for DENTAL REGISTER by "Mrs. M. W. J.")

The Georgia State Dental Society convened in Macon, Ga., May 11, 1886.

Prof. J. H. Coyle, of Thomasville, Ga., presided.

An address of welcome was delivered by Dr. W. W. Lord, on behalf of the Macon Dental Society, and was gracefully responded to, on behalf of the State Society, by Dr. B. H. Patterson, of Baxley.

The President's Annual Address was a masterly paper, on the subject of Dental Education.

He said this is the great question for Association work, all others being mere incidental outgrowths. The extension of knowledge in its practical bearings on the profession overshadows every other consideration. The Georgia State Dental Society, through its Examining Board, had been armed by the State with authority to stand as a stone wall between the people and empiricism in the fight against ignorant bushwackers and licensed ignorance. He emphasized strongly the necessity for a higher education. The competent dentist must understand the anatomy of the organs upon which he operates; without a knowledge of pathology of function and physiology, he cannot understand morbid conditions; without some knowledge of chemistry he can not comprehend the reactions in the oral cavity; a pretence to prophylaxis would be an absurdity without a knowledge of etiology; he must have some knowledge of materia medica for the application of therapeutics.

Prof. Coyle then gave a history of the successive steps through which the present law had been secured, especially the enactment of a recent amendment, which requires, not only that the practitioner shall hold a diploma, which might possibly be obtained without proper qualifications, but that, before receiving a license to practice in the State of Georgia, he shall satisfactorily pass an examination by the State Board of Examiners.

He said that to such action on the part of State Societies was due the real reforms in Dental Collegiate Education, and the organization of the National Association of Dental Faculties, with the demand for rigid examination both on entering college, on promotion to higher classes, and at graduation; with the absolute demand for two full winter courses, practically revolutionizing the standard of collegiate attainments.

He then spoke of the possible tendency of the present Georgia State law to discourage collegiate education, a license obtained from the Georgia Board being practically equivalent to a Diploma in that State. Students might possibly suppose that they could obtain the necessary qualifications to pass the Board, in a private office and by home reading, at less expense and in less time than by attending college. Not that this could be done, but that students, and possibly some preceptors might be led into this error.

Dr. Coyle paid a feeling tribute to the memory of Drs. John P. Holmes, M. S. Jobson, and N. O. Best, deceased during the past year.

He urged upon each member of the society the duty of coming forward and contributing his mite, putting aside sloth and false pride, and while drinking from the fountain of the experience of others, letting all discussions be tempered with charity and polished by courtesy.

The address was received with warm applause, and on motion, made the subject of discussion.

The point especially discussed was the fears expressed by the President that the State Board might constitute itself a Dental College, in the exercise of its legal powers.

*Dr. Catching* said that the original dental law of Georgia enabled any man holding a diploma to practice dentistry in the State. The Delavan College and other similar institutions having flooded the country with bogus diplomas, something more had been found necessary for the protection of the people from quackery. He thought the result of the recent amendment to the law would be to stimulate the colleges and raise the standard of graduation, as only those thoroughly competent could hope to pass the rigid examination exacted by the State Board of Dental Examiners.

*Dr. L. D. Carpenter* thought that it would also stimulate students. They would soon learn that they needed the very best education they could get at the very best dental college to enable them to pass a Board which unhesitatingly rejected all who were found incompetent.

*Dr. W. W. Ford* said that under the old law, any student who obtained a diploma, perhaps by two weeks attendance during commencement, and paying the necessary fee, could snap his fingers at the Georgia State Board, but it would now be found a very different matter.

*Dr. R. B. Adair* thought rejection by the Board would prove a most salutary lesson both to colleges and to students. The privilege of the floor having been extended to visiting dentists, *Dr. J. R. Walker*, New Orleans, said that it was not possible for any private preceptor to offer the advantages of a properly equipped dental college.

*Dr. Ford* said it was well known that the Dean of the Baltimore College advocated the rule adopted by the Johns-Hopkins University, viz.: If a man knows a thing, allow him the use of it; if he can learn it in a month, don't hold him back a year.

*Prof. Coyle* said the principle adopted by the Johns-Hopkins University was not to ask a man on entering, "where and how long it took him to learn a thing," but, "what do you know?" It has been found that, in a dental college, this test, however rigidly enforced, had nevertheless opened the door to rascality—men had been turned loose upon the community without proper qualifications. The National College Association had been organized with a view to correct these evils, and he hoped it would be sustained by the State Associations. It was necessary to have perfect unanimity of action in this matter.

If students got the impression that they could practice in Georgia without a diploma, by getting a license from the State Board, they would attempt to prepare themselves for the examination by private instructions. Of course, as a rule, they would meet only with disappointment and rejection. But there was a discrepancy somewhere. According to the law it was all right for the Board to give a license to practice, on the ground of a man's acquirements; but that it was all wrong for a college to give a diploma with less than two full courses of lectures. Was the Board less liable to be irresponsible or corrupt than the College Faculty?

A thorough education in dentistry required the apparatus and facilities of a well equipped dental college. The subject was passed.

*Dr. J. L. Fogg* read a paper on

### DENTAL PATHOLOGY.

Pathology is the study of the cause of disease, Surgery a curative art, its object being the treatment of external disease, injuries, and malformations. His special subject was the abnormal osseous deposit around and in human teeth, exostosis and nodules. He considered them to be the abnormal results of efforts of nature to repair some injury or heal some inflammatory troubles; or an excess of nourishment carried to the roots by the periosteum, or to the pulp by the blood vessels—an excess over what was required, and favored by constitutional idiosyncracies. There was no sure cure for such hypertrophy but extraction for the one and devitalization for the other. The extraction of exostosed teeth was usually very difficult. He preferred to drill down by the side of the root with cone-shaped engine point, enlarging the socket, and removing with narrow beak forceps, as leaving the parts in better condition for healing than if removed with incising forceps. For the removal of nodules he would drill a pocket in direct line with the nerve canal.

*Dr. Ford* regretted that the paper did not suggest any remedy for the disease exostosis. He reported an extreme case, in which all of the upper teeth had to be cut out to the very apex, being as brittle as glass, and the nerve canals entirely obliterated. Extraction and the immediate insertion of a rubber plate were followed by extreme and rapid absorption, the mouth changing so rapidly that eight plates were made in as many years. The last plate made was a hollow shell of gold, with teeth an inch and a quarter long. No absorption followed the insertion of a gold plate.

*Dr. R. B. Adair* spoke of the difficulty of diagnosing exostosis, except in extreme cases where there was some enlargement perceptible through the alveolus.

*Dr. B. H. Catching* asked for information as to the cause of deep grooves sometimes found on the buccal surface of the teeth, worn as though sawed with a string.

*Dr. Walker* attributed it to the lateral motion of a very stiff brush, very persistently used.

*Dr. H. A. Lowrance* had seen the same thing in the mouth of a patient who had perhaps never used a tooth-brush.

*Dr. Ford* said that where the teeth were very sensitive from that or other cause, he recommended the free use of lime water as a wash usually with great benefit.

*Dr. Walker* thought the lime water would be beneficial where the trouble was due to the action of acids, and also, by hardening the teeth preventing further mechanical attrition. Subject passed.

*Dr. Parsons*, who was unable to attend, sent a paper on

MATERIALS FOR FILLING TEETH, which was read by the President.

*Dr. Parsons* spoke of the different forms of *gold* in use, placing them all in two classes—soft or non-cohesive and cohesive. He said that the successful use of gold required not only skill on the part of the operator, but also good tooth substance to work upon. Vast numbers of teeth that could not have been saved when gold was the only material relied upon, were saved now by the use of other materials which were better adapted to the conditions of tooth structure. Sometimes, however, when the operator was skilled, and the gold good, and the tooth good, an operation would fail, from other circumstances.

Next to gold he classed *amalgam*, which has now nearly reached the stage of perfection, and is largely used by the majority of dentists the world over, proving a God-send to those who have not the means to pay for gold. Where the cavity is as thoroughly prepared as for gold, it makes a good permanent filling.

Experiments are still being made in the endeavor to procure a *cement* which shall resemble enamel in texture and color, crystalize without shrinking and preserve the tooth as well as gold. All that could be desired has not yet been accomplished, but what we have will save some teeth that could not be saved with either gold or amalgam. The oxy-chloride cements are escharotic —the oxy-phosphates non-irritant. The latter is very valuable where the dentine is very sensitive, caused by inflamed tubuli. The cements are invaluable for root fillings, and for a foundation in very large cavities, finishing with gold ; also for capping exposed nerves.

The cements are generally preferred to gutta-percha, of which the original was known as Hill's Stopping, and is good only for temporary, or experimental work. No one material is best in all cases. The interests of the patient, the constitution of the tooth, must be considered. The value of the tooth for mastication, for articulation, and for appearance is daily being more and more realized by the people.

A prominent duty of the profession is to educate the people on the value of the teeth, and the means of preserving them. The plastics have been much abused by poor operators, but that does not detract from their real value. They should be used, not abused.

This paper was discussed at some length, being extended to cover the field of Operative Dentistry.

*Dr. W. G. Browne* was surprised to hear such commendation of the plastics from Dr. Parsons, whose successful use of gold was well known. He had recently seen a number of gold fillings which were put in by Dr. Parsons forty-one years ago, and which were all in perfect condition yet.

*Dr. W. W. Ford* agreed with Dr. Parsons in the order of priority assigned to the filling materials.

Amalgams should be thoroughly tested, not only on the bench, but in the mouth, for shrinkage, discoloration, and durability, and one fixed upon, as success was more likely when the manipulation of a certain article was familiar. There were no two alike, though all composed of the same common ingredients, tin and silver, alloyed with mercury—the addition of gold, platinum, etc., was a mere catch-penny dodge, adding nothing to the value.

*Dr. W. G. Browne* used velvet cylinders almost exclusively. It was a form of gold which could be used either soft or cohesive; he used it soft, driving in an occasional cohesive cylinder, and finishing with cohesive for contour.

*Dr. J. R. Walker* said the opinions of the profession had changed much in late years on the question of *the best material*. Conditions must be the guide. Gold was best only for the best quality of teeth, with the best surroundings. For soft, decalcified teeth, he uses the cements, which exert a chemically preservative effect upon the tooth, renewing as often as necessary, especially for visible fillings in the front teeth, where it is much less perceptible than gold. For very large cavities in molars he uses amalgam, or if the tooth is sensitive, the oxy-phosphate of zinc, with gold or amalgam for masticating surface. He does not take out any excess of mercury from an amalgam, but prefers to add more material if necessary, using it as dry as possible ; used in that way it is very durable and free from shrinkage or discoloration. Chlor-percha is of great value for root fillings, and for flowing over nearly exposed nerves.

*Dr. W. F. Tignor* thought that gutta-percha would be liable to expand and cause pressure on the nerve. He caps with lead or tin, and applies a paste of oxy-phosphate of zinc and carbolic acid, filling with amalgam.

*Dr. Ford* uses as capping, one, two, or three layers of the Asbestos Felt, which comes in the box, with Pierce's oxy-phosphate, dipping it in a mixture of one part each of iodine, chloroform, and carbolic acid crystals dissolved in ten parts water, filling over it with amalgam, or with oxy-phosphate, finished at the surface with gold or amalgam.

*Dr. B. H. Catching* remarked that Dr. Parsons had not mentioned tin-foil, a filling material whose value was not sufficiently appreciated. He would never cap a pulp after the tooth had ached, if exposed through caries. If accidentally exposed in preparing a cavity, would fill with chlor-percha, or with oxy-phosphate over asbestos.

*Dr. W. G. Browne* caps with gold-book tissue paper saturated with carbolic acid, sprinkling the oxide powder over it.

*Dr. Walker* said he would take every chance of saving a nerve. If it died, the tooth was no worse off than before, the patient being warned to return on the first sympton of trouble.

*Dr. B. H. Catching* read a paper on

PATENTS AND SECRET PREPARATIONS IN DENTISTRY, which did not elicit much discussion the Society being unanimous in the condemnation of the use of anything of which the formula was not known.

*Dr. R. B. Adair* read a paper entitled

DENTAL JURISPRUDENCE.

He took the ground that there should be a fixed standard governing the civil liabilities of dentists as well as of physicians and surgeons which he thought would be very effective in doing away with quackery and irresponsible practitioners. He quoted as follows, from the charge of Judge McAdams, of the Supreme Court in a suit for damages for malpractice: "The law holds that no person should assume extra responsibility in the management of any case without bringing to the same a corresponding amount of skill to meet emergencies. The same holds true regarding every step in any operation, the surgeon being bound to exercise his best jndgment, his most watchful care, and his greatest skill for the benefit of his helpless and trusting patient."

*Dr. Adair* held that judging from this, the dentist should be held liable for any operation in dentistry unskillfully or negligently performed—held liable for all injury, unnecessary pain or suffering fairly due to his own unskillfulness, inexperience, or incompetency.

*Dr. J. M. Mason*, Chairman of the Committee on

MECHANICAL DENTISTRY

made a verbal report, which was discussed in connection with Dr. Adair's paper, the discussion of the latter having brought up the subject of damages caused by absorption through the use of rubber plates, and how far the dentist should be held liable for such damages.

*Dr. Walker* held that every dentist was presupposed to bring to bear on every operation all the ability and the skill necessary for its proper execution; that it was his duty to instruct his patients as to what was for their good. That he should be held as strictly liable for malpractice as the physician.

*Dr. T. M. Allen* did not think there was any standard by which malpractice could be judged, or by which dentists could be recognized as experts.

These questions were discussed briefly by Drs. Allen, Adair, Ford, Burt, and others.

*Dr. E. F. Adair* read a short paper on

ARRESTING CARIES

which brought out some discussion on the subject of the different methods of separating, heroic or quick wedging, being considered less liable to create inflammation and congestion than the slow gradual process of expansion. The file was emphatically condemned by all.

*Dr. R. B. Adair* thought that Perry's Separators appeared to promise all that was wanted being easily and quickly applied, and exerting their force gently.

*Dr. G. W. McElhaney* read a paper on

ALVEOLAR ABSCESS—ITS PREVENTION AND CURE.

He said that great as had been the advance of the profession in other directions, but little had been discovered on this subject since Kocker wrote sixty years ago concerning the meandering ways of an abscess, just about as it is described to-day.

*Dr. McElhaney* described briefly the usual course of an alveolar abscess, from the death of the pulp, its decomposition, the formation and escape of pus, and its various outlets, the separation of the periosteum, the generation of the sac, etc.

As preventives he named cupsicum bags, or the application of "tincture of iodine, aconite, and chloroform, equal parts," with hot foot-baths and broken doses of salts. Treatment must vary according to the temperament and health of the patient and character of tooth substance. The old method was to heal the abscess before filling, treating with creosote, carbolic acid, etc. The most approved method now is to restore the tooth to septic conditions, and fill, treating through the fistulous opening, using Farrar's Abscess Syringe. The profession is indebted to Dr. Harlan, of Chicago, for the introduction of some valuable remedies, as eugenol, sanitas oil, resorcin, per oxide of hydrogen, which offer a large field for choice, and are less disagreeable to the patient, as well as more efficacious than the old stand-bys, carbolic acid and creosote.

The subject was briefly discussed, Dr. C. T. Osborn preferring resorcin and glycerine; Dr. W. G. Browne, listerine; Dr. R. W. Thornton had successfully treated a chronic case; after failing used carbolic acid, creosote, etc., with chloral hydrate, 8 grs. to 1 oz. water, used with the hypodermic syringe. Dr. B. H. Catching and Dr. McElhaney use per oxide of hydrogen.

*Dr. W. T. Tignor* read a paper on

ANESTHESIA IN DENTAL PRACTICE.

*Dr. L. D. Carpenter* made a brief verbal report on the subject assigned him,

ARTIFICIAL DENTURES—WHAT IS BEST, leaving the choice to be decided in each case by circumstances and conditions.

*Dr. W. W. Ford* said he did not know enough, or think enough of bridgework to prepare the paper which had been assigned him. From what he did know of it, he thought a gold plate as cheap and in every way preferable.

*Dr. B. H. Catching* read a paper on

EXSECTION OF DENTAL NERVE, the record of two recent cases in his practice, successfully relieving excruciating pain which had failed to yield to all other methods of treatment. (See Southern Dental Journal, May issue.)

The election of officers was the special order of business at the last session, with the following result:

President, Dr. C. T. Osborn, Columbus.
First Vice-President, Dr. H. B. Patterson, Baxley.
Second Vice-President, Dr. W. G. Brown, Atlanta.
Recording Secretary, Dr. W. L. Smith, Hawkinsville.
Corresponding Secretary, Dr. L. D. Carpenter, Atlanta.
Treasurer, Dr. H. A. Lowrance, Athens.

The last three were re-elected.

The Committee on Memorials reported suitable resolutions on the death of Dr. A. H. Best, of Savannah, and Dr. J. P. Holmes, of Macon.

Dr. Catching proposed the establishment of a dental museum for the Georgia State Society, and Dr. W. G. Browne was appointed a committee of one to receive all contributions suitable for such a collection.

Dr. Walker called the attention of the Society to the subject of the constitutional decalification of the teeth and their recalification by systemic treatment, and requested correspondence on the subject.

Teeth are met with in the practice of every dentist that at one time are so soft and decalcified as to admit only of plastic fillings, while at another time they will have become hard and well crystalized and ready to be filled with gold with perfect safety. In the one case the lime salts have been dissolved out and carried off; in the other, there has been a more recent deposit of inorganic matter.

*Dr. Tignor* said that when such changes occur, it must be in obedience to fixed law, chemical or physical; nothing happens by chance.

On motion, adjourned.

